# DESCRIBING ADVANCED PRACTICE REGISTERED NURSES’ INFLUENCE ON IMPROVED NURSING HOME OUTCOMES

**DOI:** 10.1093/geroni/igae098.4363

**Published:** 2024-12-31

**Authors:** Alisha Johnson, Amy Vogelsmeier, Veronica Thompson

**Affiliations:** University of Missouri, Columbia, Missouri, United States; University of Missouri, Columbia, Missouri, United States; University of Missouri, Columbia, Missouri, United States

## Abstract

Advanced practice registered nurses (APRN) embedded full-time within nursing homes (NH) improve key resident outcomes such as reducing hospitalizations, increasing advance directive use, and improving resident/family satisfaction. Despite positive evidence, this care model is not widely adopted, and there has been minimal research on how APRN practice impacts outcomes. Informed by Donabedian’s Structure-Process-Outcome framework, we conducted a cross-case analysis to understand how APRNs influenced patient (direct) and facility-level (indirect) outcomes through nursing home structures and processes. We used eight years of data generated from The Missouri Quality Initiative, a CMS-funded demonstration project aimed to reduce avoidable hospitalizations through embedded APRN care in 16 midwestern nursing homes. Cases consisted of two higher-performing homes and two lower-performing NHs. Each case was analyzed using deductive coding of four databases to create a cross-database table. First and second level thematic coding was applied to the cross-database tables to create the high and low performing cases for cross-case comparison. Stark differences arose between the higher and the lower performing cases around themes including proactive/reactive processes, individual vs. group-oriented processes, and administrative supports (present/absent/lip-service). APRNs applied proactive processes at the patient (direct) and facility (indirect) level when NH structure (i.e. adequate administrative support) was present. More successful homes, even with higher turnover, demonstrated proactive mentoring processes, initiated and supported by the APRN, instead of passive, reactive processes and deference to administration. APRNs are effective caregivers if structural supports allow for proactive processes maximizing APRN expertise, supporting the inclusion of APRNs within NH care delivery models.

